# The Representation of Motor (Inter)action, States of Action, and Learning: Three Perspectives on Motor Learning by Way of Imagery and Execution

**DOI:** 10.3389/fpsyg.2017.00678

**Published:** 2017-05-23

**Authors:** Cornelia Frank, Thomas Schack

**Affiliations:** ^1^Neurocognition and Action – Research Group, Faculty of Psychology and Sports Science, Bielefeld UniversityBielefeld, Germany; ^2^Cognitive Interaction Technology – Cluster of Excellence, Bielefeld UniversityBielefeld, Germany; ^3^Research Institute for Cognition and Robotics (CoR-Lab), Bielefeld UniversityBielefeld, Germany

**Keywords:** motor imagery, motor memory, simulation, s-states, intelligent systems, functional equivalence

## Abstract

Learning in intelligent systems is a result of direct and indirect interaction with the environment. While humans can learn by way of different states of (inter)action such as the execution or the imagery of an action, their unique potential to induce brain- and mind-related changes in the motor action system is still being debated. The systematic repetition of different states of action (e.g., physical and/or mental practice) and their contribution to the learning of complex motor actions has traditionally been approached by way of performance improvements. More recently, approaches highlighting the role of action representation in the learning of complex motor actions have evolved and may provide additional insight into the learning process. In the present perspective paper, we build on brain-related findings and sketch recent research on learning by way of imagery and execution from a hierarchical, perceptual-cognitive approach to motor control and learning. These findings provide insights into the learning of intelligent systems from a perceptual-cognitive, representation-based perspective and as such add to our current understanding of action representation in memory and its changes with practice. Future research should build bridges between approaches in order to more thoroughly understand functional changes throughout the learning process and to facilitate motor learning, which may have particular importance for cognitive systems research in robotics, rehabilitation, and sports.

## Introduction

Learning in intelligent systems is a result of direct and indirect interaction with the environment. To understand how intelligent systems learn to adequately act in a given environment with respect to a particular task, thereby adapting, is of particular relevance to cognitive science disciplines such as psychology, biology, and computer science (e.g., Pfeifer and Bongard, 2007; Wolpert et al., 2011; Abrahamsen and Bechtel, 2012; Pacherie, 2012; Engel et al., 2013, 2015). This capability of goal-directed motor (inter)action changes and develops with practice, transitioning from unskilled into skilled motor (inter)action, and resulting in refined planning and execution of motor (inter)actions (e.g., Meinel and Schnabel, 2007; Schmidt and Wrisberg, 2008; Magill, 2011; Schmidt and Lee, 2011). Interestingly, advancing our understanding of intelligent systems’ actions and their acquisition remains a significant endeavor to this day, especially in view of prospective applications in various settings such as robotics, psychology, sports, and rehabilitation. For instance, the development of intelligent interactive technical platforms which are to assist humans requires a thorough understanding of natural, intelligent forms of (inter)action and their acquisition, respectively (e.g., Pfeifer and Bongard, 2007; Schack and Ritter, 2009, 2013; Di Nuovo et al., 2013; De Kleijn et al., 2014). Understanding learning by way of different states of action (e.g., imagery or execution) and related functional changes within the motor action system, particularly with regards to action representation may help to advance in this direction. Here, we overview the literature on learning by imagery and execution from three perspectives, namely the performance, the brain, and the mind perspective.

## States of (Inter)Action and Learning

An action reflects “a set of mechanisms that are aimed at producing activation of the motor system for reaching a goal” (Jeannerod, 2004, p. 376). Similarly, interaction may be considered as sets of mechanisms of several individuals acting together, which are aimed at producing activations of all motor systems involved for reaching a shared goal. (Inter)actions can overt as well as covert actions, that is executed, imagined or observed actions (Jeannerod, 2001, 2004). Given the principle of functional equivalence (Finke, 1979; Johnson, 1980; Jeannerod, 1994, 1995) and the simulation theory (Jeannerod, 2001, 2004, 2006), executed, imagined, and observed actions are all suggested to be actions, as each draws on the same action representation. While ‘actual’ actions involve both a covert (e.g., planning) and an overt (e.g., execution) stage of action, ‘simulated’ actions such as imagery imply a covert stage of action only (i.e., simulation state; s-state; Jeannerod, 2001). To this extent, each of the different types of s-states to some degree involves the activation of the motor action system. That is, any form of executed or simulated state of action is considered an action, regardless of whether it includes covert stages of action only or both covert and overt stages of action. Given the principle of functional equivalence, the repeated use of any of these states as means of practice should lead to functional changes within the motor action system and to learning. Accordingly, mental types of practice have been suggested to be effective means to induce learning (e.g., Jeannerod, 1994, 1995, 2001, 2004).

To date, it is widely accepted that humans can learn by way of different states of (inter-)action, but their unique potential to induce changes in the motor action system is still being debated (e.g., Driskell et al., 1994; Allami et al., 2014; Di Rienzo et al., 2016; Frank et al., 2016). Interestingly, while evidence on the functional equivalence of executed and imagined actions is vast (e.g., Finke, 1979; Johnson, 1980; Jeannerod, 1994, 1995, 2001; Decety, 1996, 2002; Jeannerod and Frak, 1999), only little is known about how learning by execution or imagery works. Furthermore, it is unclear what the similarities and differences of these ways of learning are, particularly with regards to changes in action representation. In other words, research has yet to systematically examine the differential effects of learning by way of different states of action.

In this perspective paper, we focus on learning by way of imagery and execution, and discuss it from a perceptual-cognitive point of view on action representation. For this purpose, we review learning by way of imagery and execution from three different levels of analyses. First, we examine the literature from the performance perspective (here: in terms of changes in motor behavior), followed by the brain perspective (here: in terms of changes in neurophysiological representations of motor action), and finally by the mind perspective (here: in terms of changes in perceptual-cognitive representations of motor action). In doing so, we highlight the role of action representation within a motor hierarchy, and exemplify how such models could advance our understanding of learning, enabling links between neurophysiological approaches and motor control and learning theories. Finally, we discuss potential future directions to advance research comparing learning by way of execution, imagery, and other states of action.

## The Performance Perspective on Imagery and Execution: Learning as Changes in Motor Performance

The systematic use of different states of action for practice and their contribution to the learning of complex motor actions has traditionally been approached by way of persisting performance improvements (e.g., Schmidt and Lee, 2011). Similarly, researchers investigating the influence of mental practice traditionally have focused on motor performance (e.g., Corbin, 1967a,b; for reviews and meta-analyses, see Richardson, 1967a,b; Feltz and Landers, 1983; Feltz et al., 1988; Hinshaw, 1991; Grouios, 1992; Driskell et al., 1994). From this, mental practice has shown to be more effective than no practice, but less effective than physical practice (e.g., Feltz and Landers, 1983; Feltz et al., 1988; Driskell et al., 1994). Driskell et al. (1994), for instance, conducted a meta-analysis on the effects of mental practice in comparison to irrelevant practice and physical practice, reporting an overall average effect size of *d* = 0.53^1^ for mental practice, and an effect size of *d* = 0.78 for physical practice. Moreover, combined mental and physical practice has been suggested to be as effective as or superior to physical practice (e.g., Corbin, 1967b; McBride and Rothstein, 1979; Hall et al., 1992; Gomes et al., 2014). From this perspective, mental practice is considered a potentially effective means to promote learning.

## The Brain Perspective on Imagery and Execution: Learning as Changes in Neurophysiological Action Representation

In search of answers to the question why learning by way of different states of action works (e.g., Heuer, 1985; Murphy, 1990; Murphy et al., 2008), neurocognitive approaches have evolved, considering learning from within (e.g., Jeannerod, 2001, 2004). Neurocognitive approaches highlight the role of action representation in the learning of complex motor actions from a neurophysiological perspective. So far, the adaptation of the brain (i.e., neurophysiological and -anatomical changes) as a result of physical practice has received a great deal of attention (e.g., Wadden et al., 2012). From this, multifaceted insights into central changes within the motor action system have been provided regarding the neural aspects of learning a motor action, and the neural plasticity of the brain, respectively (for a recent meta-analysis, see Hardwick et al., 2013; for reviews, see also e.g., Doyon and Ungerleider, 2002; Ungerleider et al., 2002; Doyon and Benali, 2005; Kelly and Garavan, 2005; Halsband and Lange, 2006; Dayan and Cohen, 2011). In the context of the principle of functional equivalence and the simulation theory (Jeannerod, 2001, 2004, 2006), the study of action representation from a neurophysiological point of view has received tremendous research interest (for overviews, see e.g., Decety, 2002; Guillot et al., 2014). While considerable research attention has been directed to comparing the different states of action, such as the imagery and the execution of an action (e.g., Decety, 1996, 2002; Jeannerod and Frak, 1999), only few studies exist that compare learning by way of imagery and execution and respective changes in the brain (e.g., Pascual-Leone et al., 1995; Jackson et al., 2003; Nyberg et al., 2006; Zhang et al., 2012, 2014; Allami et al., 2014; Avanzino et al., 2015; for a review, see Di Rienzo et al., 2016).

For instance, Pascual-Leone et al. (1995) investigated plastic changes in the human motor action system resulting from physical and mental practice, using transcranial magnetic stimulation. Interestingly, while the authors found physical practice to be superior to mental practice in terms of performance improvement in a key pressing task, both physical and mental practice led to the same plastic changes, namely an equally increased size of the cortical representation for the finger muscle groups involved. From this, the authors concluded that mental practice modulates the neural circuits involved in learning, potentially by forming a cognitive model of the motor action. Jackson et al. (2003) investigated cerebral functional changes in the brain as induced by mentally practicing foot movements employing positron emission tomography and compared these changes to those induced by physically practicing foot movements (Lafleur et al., 2002). Similar to the findings reported by Lafleur et al. (2002) on physical practice effects, the authors found mental practice to be associated with functional cerebral reorganization in the right medial orbitofrontal cortex. From the lack of striatum activation after mental practice, however, the authors suggest that the re-organization rather relates to the planning and the anticipation of motor actions than to its motor execution. More recently, Zhang et al. (2014) examined changes in functional connectivity in resting state as a result of mental practice, using functional magnetic resonance imaging. The authors reported alterations in cognitive and sensory resting state networks in various brain systems after learning by way of motor imagery (i.e., mental practice), while no alterations in connectivity were found in the control condition (i.e., no practice). From this, the authors concluded that modulation of resting-state functional connectivity as induced by mental practice may be associated with attenuation in cognitive processing related to the formation of motor schemas. These neurophysiological studies on learning as induced by mental practice and/or physical practice show that both mental and physical practice lead to significant changes in action-related brain activation during skill acquisition. At the same time, however, they reveal distinct differences pointing to a hierarchy in learning by way of different states of action (for more details, see discussion section).

From a neurophysiological perspective, learning can be considered as neurophysiological reorganization, with the neurophysiological representation of motor action functionally developing over the course of the learning process. This seems to hold for both learning by execution and learning by imagery. Neurophysiological studies as the ones exemplified above provide valuable multifaceted insights into the functional changes in brain activation as a result of physical and mental practice. Findings elucidating neurophysiological changes associated with motor learning as induced by mental and physical practice, however, do not necessarily allow for specific conclusions regarding action representation and its relation to motor control. Therefore, it seems important to link these approaches to models and theories of motor control and learning, particularly those emphasizing the role of action representation, in order to be able to draw specific conclusions about changes of the motor action system during learning. To put it differently: Given the functional reorganization of neurophysiological features in the brain, is there a functional reorganization of perceptual-cognitive representations of motor (inter)action in the mind as part of a functional stratification on various levels within the motor action system?

## The Mind Perspective on Imagery and Execution: Learning as Changes in Perceptual-Cognitive Action Representation

According to perceptual-cognitive approaches (e.g., theory of anticipative behavioral control: Hoffmann, 1993; theory of event coding: Hommel et al., 2001; simulation theory: Jeannerod, 2001) and the original idea of a bidirectional link between an action and its effects (i.e., ideomotor theory: James, 1890), actions are primarily guided by cognitively represented perceptual effects. Drawing on the seminal work of Bernstein (1967) and his idea of a model of the desired future, motor actions can be considered as being stored in memory as well-integrated representational networks or taxonomies comprised of perceptual-cognitive units (i.e., basic action concepts; BACs) that guide action execution (cf. cognitive action architecture approach/ CAA-A: for an overview, see Schack, 2004; Schack and Ritter, 2009). Moreover, these networks of BACs are suggested to change throughout the process of motor learning by way of perceptual-cognitive scaffolding, resulting in a more elaborate perceptual-cognitive representation.

Based on research relating to CAA-A (e.g., Schack and Mechsner, 2006), experts, as compared to novices, have been shown to hold structured representations. A functionally structured representation is comprised of groupings of perceptual-cognitive units (i.e., groupings of BACs) that relate to the same (sub-)functions of the action, and thus reflect the functional phases of the motor action (cf. Göhner, 1992, 1999; Hossner et al., 2015). Schack and Mechsner (2006), for instance, examined representational networks of the tennis serve in experts and non-experts, using the structural dimensional analysis of mental representations (SDA-M). Results elicited that skilled individuals held functionally structured representations relating well to the biomechanical demands of the task (i.e., reflecting clearly the three movement phases pre-activation, strike, and final swing), whereas unskilled individuals’ representations were unstructured. This has been shown to generalize to motor skills of different complexities (e.g., manual action: Braun et al., 2007; gait: Schega et al., 2014; Stöckel et al., 2015; dance: Bläsing, 2010).

With regards to learning, action representations have been shown to functionally adapt in the direction of an elaborate representation during motor learning (Frank et al., 2013). Findings revealed that, together with improvements in golf putting performance, representations changed with practice, developing toward more functional ones, with groupings of perceptual-cognitive units (i.e., groupings of BACs) relating more closely to the same (sub-)functions of the action itself (i.e., preparation, forward swing, and impact). Drawing on the finding that novices’ perceptual-cognitive representations of complex action develop and adapt with practice, Frank et al. (2014) addressed the development of one’s representation according to type of practice, comparing physical practice (i.e., repeated motor execution), mental practice (i.e., repeated motor imagery) and their combination. While motor performance reflected the well-known pattern of magnitude of improvement according to type of practice (i.e., combined practice > physical practice > mental practice > no practice), mental practice, either solely or in combination with physical practice, led to even more elaborate representations compared to physical practice only. Representation structures of the groups practicing mentally became more similar to a functional expert structure, whereas those of the physical practice group revealed less development. Building on these findings, Frank et al. (2016) further examined the perceptual-cognitive background of performance changes that occur within the motor action system as a result of mental and physical practice, employing a mobile eye-tracking system to investigate gaze behavior (i.e., the quiet eye; e.g., Vickers, 1992, 1996, 2009). Combined practice led both to more developed representation structures and to more elaborate gaze behavior prior to the execution of the putt, with final fixations prior to the onset of the putting movement (i.e., the quiet eye) being longest for this group and better developed representation structures relating to longer quiet eye durations after learning. Accordingly, the quiet eye might reflect a predictive mode of control that initiates a cognitively demanding process of motor planning based on the representation available (for details on a perceptual-cognitive perspective on the quiet eye, see Frank and Schack, 2016).

More recently, learning as it relates to interaction was investigated by examining representational frameworks of interaction and their development with mental practice (Frank et al., under review). The impact of a team action imagery intervention on futsal player’s shared representations of team-specific tactics was investigated. Mental practice consisted of practicing four team-specific tactics (i.e., counter-attack, play making, pressing, transitioning) by imagining team actions in specific game situations for three times a week over the course of 4 weeks. Results revealed representational networks of team action becoming more similar to those of experts after mental practice. This study indicates that the imagery of team actions can have a significant impact on players’ representational networks of interaction in long-term memory.

From this line of studies, the learning of a motor action can be considered as perceptual-cognitive reorganization, with the perceptual-cognitive representation of action functionally developing throughout the learning process. This research furthermore indicates that the perceptual-cognitive reorganization taking place during learning depends on the state of action used for practice. Learning by way of imagery differs from learning by way of execution, with practice through imagery promoting the functional development of a perceptual-cognitive action representation (perceptual-cognitive explanation of mental practice), while not necessarily transferring one-to-one to motor performance. This points to a differential influence of mental and physical practice with regards to different levels of action organization, with mental practice operating primarily on higher levels within the motor action system, particularly during early skill acquisition (for a more detailed discussion, see Frank, 2014). This approach, particularly together with neurophysiological approaches, may add to the picture of potential basic mechanisms that underlie each type of practice, an issue still being highly debated (e.g., Annett, 1995; Jackson et al., 2001; Munzert et al., 2008; Murphy et al., 2008; Cumming and Williams, 2012; Glover and Dixon, 2013). By complementing existing evidence from a performance and a brain perspective on learning by mental and physical practice (e.g., Driskell et al., 1994; Allami et al., 2014), these findings contribute to a better understanding of the adapting motor action system, by disentangling changes on various levels within the motor action system during learning.

## Discussion and Conclusion

While there is ample evidence on the functional equivalence between different states of action (such as the imagery and the execution of an action; e.g., Decety, 1996, 2002; Jeannerod and Frak, 1999), research addressing the similarity or difference with respect to the influence that each of the states of action has on the motor action system during learning has remained scarce to date. Meanwhile, more and more researchers have claimed to take into account potential differences between the states of action and their contribution to motor control and learning, as these might be as well (or in particular) meaningful to fully understand the motor action system (e.g., Munzert et al., 2009; Wakefield et al., 2013; O’Shea and Moran, 2017). Given that each state of action differs to some degree, the repeated use of imagery or execution is likely to differ in their influence on the motor action system. In other words, while the repeated use of imagery and execution of an action is suggested to result in learning, learning is likely to differ as a function of the state of action used for practice.

Here, we outlined learning by way of imagery and execution from three perspectives. While there is ample evidence from the performance perspective (for a review, see e.g., Driskell et al., 1994), the research from a brain perspective (for a review, see e.g., Di Rienzo et al., 2016), and from a mind perspective (e.g., Frank et al., 2016) on action representation as it relates to learning by imagery and execution has just started to gain momentum. Despite these initial steps, the potential of imagery and execution to induce changes within the motor action hierarchy during learning, however, remains to be explored more thoroughly. Interestingly, although sometimes not explicitly introduced as the theoretical background of their studies, (indirect or direct) conclusions about the formation of action representations are drawn from the brain changes observed, linking neurophysiological findings to hierarchical motor control and learning theories: for instance, Pascual-Leone et al. (1995, p. 1043) discussed that repeated imagery may help establish a cognitive model of the motor action; Zhang et al. (2014, p. 4) state that motor schemas have developed; Jackson et al. (2003, p. 1178) discuss from the lack of striatum activation after mental practice, that the re-organization relates to the planning and the anticipation of motor actions rather than to its motor execution. By doing so, each of the studies implicitly refers to a theoretical background of motor control and learning, and alludes to some form of representational format in memory. However, the results of these studies have not yet been discussed in the light of hierarchical models of action organization, focusing on higher and lower levels of action representation, as the one delineated in the present perspective paper. By suggesting that mental practice helps promote a ‘cognitive model,’ ‘attenuated cognitive processing,’ and the ‘planning and the anticipation of actions,’ these findings are in line with the perceptual-cognitive explanation of mental practice and the idea that the repeated use of imagery particularly helps establish perceptual-cognitive representations of action (Frank et al., 2014, 2016).

Future studies may place more emphasis on the role of action representation and compare learning by way of imagery and learning by execution with regards to brain- and mind-related changes on different levels within motor action system. For instance, related research disentangling neurophysiological representations of actions within a motor hierarchy (e.g., Grafton and Hamilton, 2007), research on the degree of abstractness of neurophysiological representation of actions (e.g., Tucciarelli et al., 2015; Wurm and Lingnau, 2015; Turella et al., 2016), or research on neurophysiological representations’ structural geometry across states of action (Zabicki et al., 2016) in conjunction with perceptual-cognitive approaches to motor learning might be promising avenues to better understand learning across states of action. In a recent study, for instance, Zabicki et al. (2016) investigated imagined and executed actions using a multivariate approach and a representational similarity analysis to neurophysiological representations of action, highlighting a similar structural geometry as well as distinct differences in action representation between the two states of action. Using such approaches together with hierarchical, perceptual-cognitive ones in the realm of motor cognition might help to further approach the phenomenon of action representation in motor control and learning and the unique potential of imagery and execution to induce changes on different levels within the motor action system during learning.

In sum, research directly comparing the two modes of learning has remained scarce to date, with many studies focusing on one mode only (e.g., imagery: Zhang et al., 2014; execution: Lafleur et al., 2002). Furthermore, most of the studies conducted so far focus on the potential similarities that learning by way of motor imagery may share with learning by way of motor execution, thereby disregarding potential differences across learning types, such as a differential influence on various levels within the motor action system. And finally, the brain and the mind perspective have been considered merely isolated, investigating neurophysiological representations or perceptual-cognitive representations. Accordingly, three main challenges may have to be addressed by future studies in order to advance research comparing learning by way of execution, imagery, and observation, and thus to more thoroughly understanding intelligent systems and learning by different states of action. First, research comparing learning by different states of action should be conducted in a systematic manner, employing research designs that allow for examining states of action both in isolation as well as in combination (cf. four group design in mental practice research, e.g., Corbin, 1967b; Hall et al., 1992). Second, research questions and hypotheses should be directed toward the differences between learning by different states of action, and thus going beyond the traditional focus on the functional equivalence between the states of action, and the potential similarities across learning types, toward a hierarchical view of the motor action system. Third, learning by different states of action should be approached in future research by integrating findings and methods from different disciplines (e.g., Moran et al., 2012) such as the ones exemplified above in order to approach the problem from distinct, but complementary perspectives.

To systematically examine learning by different states of action from various perspectives focusing on both the similarities and the differences across higher and lower levels of action organization may contribute to a better understanding of the motor action system. Complementing both the performance and the brain perspective by a mind perspective may lead to advancing our understanding of intelligent systems in general, and the learning of (inter)action across states of action in particular, in order to better be able to design training tools that facilitate motor (re)learning. Future research should therefore build bridges between the perspectives in order to more thoroughly understand functional changes throughout learning across states of action, and to subsequently address specific levels within the motor action hierarchy as part of individualized coaching in robotics, rehabilitation, or sports settings (e.g., Hülsmann et al., 2016).

## Author Contributions

Conception and draft of perspective paper (CF and TS).

## Conflict of Interest Statement

The authors declare that the research was conducted in the absence of any commercial or financial relationships that could be construed as a potential conflict of interest.

## Footnotes

1 Effect sizes reported throughout this chapter refer to Cohen’s *d* (Cohen, 1992).
